# How Are School Menus Evaluated in Different Countries? A Systematic Review

**DOI:** 10.3390/foods10020374

**Published:** 2021-02-09

**Authors:** Alessandra Fabrino Cupertino, Dayanne da Costa Maynard, Fabiana Lopes Nalon de Queiroz, Renata Puppin Zandonadi, Verônica Cortez Ginani, António Raposo, Ariana Saraiva, Raquel Braz Assunção Botelho

**Affiliations:** 1 Department of Nutrition, University of Brasília, Brasília 70910-900, Brazil; acupertino1006@gmail.com (A.F.C.); day_nut@yahoo.com.br (D.d.C.M.); fabinalon@hotmail.com (F.L.N.d.Q.); renatapz@unb.br (R.P.Z.); vcginani@gmail.com (V.C.G.); 2 CBIOS (Research Center for Biosciences and Health Technologies), Universidade Lusófona de Humanidades e Tecnologias, Campo Grande 376, 1749-024 Lisboa, Portugal; 3 Department of Animal Pathology and Production, Bromatology and Food Technology, Faculty of Veterinary, Universidad de Las Palmas de Gran Canaria, Trasmontaña s/n, 35413 Arucas, Spain; ariana_23@outlook.pt

**Keywords:** evaluation, instrument, menu, school feeding

## Abstract

School meals should focus on quality of life issues, particularly on reducing food shortages, overweight, obesity and its consequences. As an essential tool for quality assurance, creating the menu is an activity of great complexity and requires multidisciplinary knowledge. This activity covers the observation of countless aspects of quality, highlighting nutritional, sensory, cultural, hygienic, and sanitary issues, among others. This study aims to identify and analyze instruments and methods to evaluate school menus in different countries. The authors developed specific search strategies for Scopus, Web of Science, Science Direct, Pubmed, Lilacs, ProQuest Global, and Google Scholar. The included studies’ methodological quality was assessed using the statistical analysis and meta-analysis review tool (MASTARI). A total of 16 cross-sectional studies met the inclusion criteria and were analyzed. Brazil and Spain were the countries that presented the highest number of studies (*n* = 5; 31.25% for each). The majority of the studies have a qualitative approach (*n* = 12, 75%), and only 25% (*n* = 4) of the studies present quantitative assessment methods to evaluate school menus. No school menu assessment tools were found to assess all aspects of menu planning. The results show a lack of a methodology or of instruments for evaluating the menus offered for school meals that can contribute to better dietary care offered to students.

## 1. Introduction

Globally, inadequate nutrition is one of the main challenges representing a threat to individuals’ health, including their well-being and productivity. This results in high socioeconomic costs for societies in all regions of the world. Therefore, early healthy eating habits are essential to reduce the risk of immediate and long-term health problems [1].

The school has a strong influence on the students’ behavior, and when their environment is adequate, it is considered favorable for the formation and consolidation of healthy habits [2]. According to the Food and Agriculture Organization (FAO), about 40 million children under five years are overweight, and about 120 million children (ages 5 to 10) and adolescents (ages 11 to 19) are obese [1]. Based on the need to change this scenario, the World Health Organization (WHO) defined in 2001 a global strategy for feeding young children, considering respect, protection, and fulfillment of human rights. It recommends that school menus prioritize promoting healthy and adequate food for children and adolescents. Thus, it is recommended to use varied and safe foods, promoting eating habits that contribute to students’ growth and development and academic performance [3].

Despite the menu’s definition as a list of dishes of a planned meal, the menu goes beyond its initial concept in the scholarly environment. It imposes itself as the vehicle to ensure that school meals meet their proposed aims. The menu may offer foods that contribute to the students’ health, considering nutritional, sensory, cultural, and microbiological aspects [4]. Therefore, the menu also contributes to the formation of healthy eating habits and the preservation of the environment and of food culture, providing nutritional education [5].

Planning and evaluating menus is essential to meet recommendations and contribute to adequate nutrition. School environments must offer healthy and nutritious meals through balanced menus. Families, educators, and governments from different countries should contribute to the formulation, implementation, monitoring, and evaluation of a comprehensive national policy on school feeding [3,6,7].

Despite the existence of criteria and different menu planning methods, there is no consensus on the best protocol for evaluating them, especially concerning school menus [8,9]. In general, in schools, a qualitative assessment of menus is prioritized. It is not clear in the literature whether the assessment instruments meet the recommendations of school feeding programs regarding all their aims, nor how the instruments are applied. It is unknown whether the existing instruments are used to evaluate the executed or planned menus [10,11].

School menu evaluation protocols are essential to ensure their planning. These protocols, defined in this study as a systematic way of evaluating menus, also need to be assessed, disseminated and implemented across different realities. Thus, it is essential to know what protocols are available, what kind of instruments or methods, and how they work, ensuring they comply with the WHO/FAO assumptions about healthy eating. This study is justified by the need to understand how school menus are planned and evaluated worldwide as they directly impact the quality of meals (breakfast, snacks, lunch and/or dinner) served in schools. Therefore, the present systematic review’s objective is to identify and analyze protocols for evaluating available school menus, with no restrictions by country for studies included in the review.

## 2. Materials and Methods

This systematic review focused on protocols for evaluating school menus worldwide. It was prepared according to the report items for systematic reviews and meta-analyses (PRISMA) and its Checklist [12,13,14,15,16]. The protocol was performed according to the following steps.

### 2.1. Eligibility Criteria

The inclusion criteria were studies of school menu assessment instruments and school menus related to governmental school programs, with no date, language, or publication status limits. The exclusion criteria applied were: (1) comments, letters, conference, review, abstracts, and books; (2) studies in private schools, unrelated to government-subsidized School Feeding Programs; (3) studies that were not performed on school food services; (4) school health programs (Table S1—Supplementary Materials).

### 2.2. Information Source

Detailed individual search strategies were developed for each database: Pubmed, Scopus, Web of Science, Science Direct, and Lilacs. A search for gray literature was carried out on Google Scholar and for dissertations and theses in ProQuest Global. The last search for all databases was carried out on 7 July 2020. Researchers carefully examined the selected study’s reference lists for full reading if any study was not retrieved during the search.

### 2.3. Search Strategy

The appropriate combination of truncation and keywords were selected and adapted for each database (Table S2—Supplementary Materials). Rayyan software (Qatar Computer Research Institute, QCRI) was used to select and exclude duplicate articles, and all bibliographic references were included using the Mendeley desktop software. Table S2 shows the search strategy used for the six databases.

### 2.4. Study Selection

The study screening process was carried out in three phases, with three independent researchers. In phase I, researchers I (A.F.B.C.) and II (D.C.M.) independently selected the articles according to their titles and abstracts. Articles that did not meet the previously established inclusion criteria were excluded. In phase II, a third independent researcher III (F.L.N.Q.) selected the conflicting articles from phase I. In phase III, the selected articles were fully read, and those that met the inclusion criteria were included. Researchers I and II critically evaluated the reference lists of the selected studies and extracted data. Other than titles and abstracts written in English, translation was performed using the Google Translator tool for studies written in other languages.

### 2.5. Data Collection Process

The selected studies’ following characteristics were collected: authors and year of publication, country of research, objective, study design, type of protocol, and school grade focus. The aspects assessed by the protocols were: nutritional, sensory, cultural, sanitary hygiene, and sustainability. Calibration exercises were performed before starting the review to ensure consistency between the two reviewers. Disagreements were solved by discussion, and the third author (F.L.N.Q.) decided on disagreements when these were not solved. These data were synthesized in a standardized table (Table 1).

### 2.6. Risk of Individual Bias in the Included Studies

The quality criteria were synthesized using a statistical review assessment instrument (MASTARI) and the Joanna Briggs Institute protocol to assess the studies’ risk of bias. The instrument for assessing the risk of bias included seven questions:

1. Were the analyzed indicators characterized?
2. Were the protocols implemented in the menu evaluation?
3. Did the evaluated indicator respond positively to implementation?
4. Is the study design adequate?
5. Was the statistical analysis adequate to the objective of the study?
6. Did the results answer the main question?
7. In the case of school mentoring services, was a sample of establishments selected to analyze the representative and randomly determined indicators?

After the analysis, the risk of bias was categorized (Table S3) as “High” when the study reached up to 49% of “yes” scores; “Moderate” when the study reached 50–69% of “yes” scores; and “Low” when the study reached more than 70% of “yes” scores.

## 3. Results

**Figure 1:**
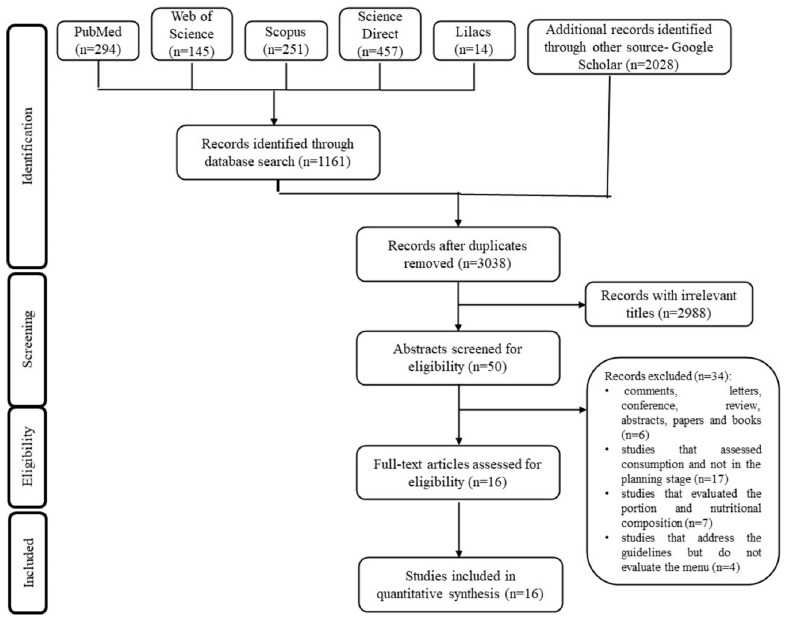
Flowchart of the systematic review search process (adapted from the PRISMA protocol).

From the 3189 studies initially selected, 52 were selected via their abstracts. After reading full texts, 16 studies met the eligibility criteria and were included in the systematic review. Figure 1 presents a flowchart of the systematic review search process.

### 3.1. Characterization of Studies

The 16 studies were from Latin America, Europe, and Australia. There is a lack of studies from North America, Central America, Asia, and Africa. The studies presented varied methods for evaluating school menus and challenges in meeting the established recommendations.

Table 1 summarizes the studies included in this review concerning the country and the type of instrument or method for menu evaluation. The studies included in the systematic review were conducted in Brazil (*n* = 5) [2,13,14,15,16]; Spain (*n* = 5) [17,18,19,20,21]; Australia (*n* = 1) [7]; Germany (*n* = 1) [22]; France (*n* = 1) [23]; England (*n* = 1) [24]; Sweden (*n* = 1) [25] and Slovenia (*n* = 1) [26], from 2009 to 2018 (Table 1).

Brazil and Spain were the countries that presented the highest number of studies (*n* = 5; 31.25% for each). In Brazil, the studies mainly discussed a checklist to evaluate the school menu (*n* = 1) [2], adequacy of serving portions meeting the premises of the national school feeding program (*n* = 1) [15], and the method of evaluating school menus by the Qualitative Analysis of Menu Preparations (*n* = 3) [13,14,16]. In Spain, a Brazilian researcher developed a study exploring a method related to the Spanish school feeding program (*n* = 1) [20]. The study was conducted in a Spanish research center. The number of studies and the public policy presence in studies of school children and adolescents justify Brazil and Spain having the same number of studies (*n* = 5; 32.25%) [17,18,19,20,21].

In Slovenia, Gregoric [26] proposed an assessment of school menus in three stages. The first was an interview with school managers, a qualitative assessment of menus via the development of a menu quality index defined by food recommendations from the National Dietary Guidelines (NDG). There is also a quantitative assessment of menus in a random sample (considering their weight and nutritional quality). For this last stage, the menus were randomly selected. The served portions were weighed, and the information on recipe, preparation, and techniques was acquired by dietitians from the Institute of Public Health using pre-established methodological instructions and ending with the calculation of nutritional composition.

In Sweden, the study was based on six domains: food groups, nutritional adequacy, food hygiene, eating behavior, sustainability, and political structure. These items were distributed across 110 questions with an organizational, pedagogical, and nutritional approach to be completed by schools. School menus can be classified as probably fulfilled, possibly fulfilled, and unlikely to comply with the recommendations. In the nutritional approach, fat, iron, vitamin D, and fibers were explicitly analyzed by adaptation to national recommendations, which could be assessed as adequate, almost adequate, or inadequate. The instrument used in this study is flexible and can be adapted to other realities, such as other countries seeking this approach model. Access to the instrument is open and available on the online platform [25].

The studies by Myers [7], Dubuisson [23], and Therre et al. [22], conducted respectively in Australia, France, and Germany, similarly approach qualitatively data on school menus in terms of variety, and quality of food, making a comparison with nutritional recommendations. They also approached food and dishes’ frequency, looking for information to better evaluate the menu’s quality.

### 3.2. School Menu Assessment Instruments

The instruments used in these studies were: a dietary questionnaire (comedores escolares—COMES) [17], EQ-MEs [19], both developed in Spain, Skolmatsverige [25] from Sweden, and CheckList [2] from Brazil. Of the instruments for evaluating the school menu, only 50% (*n* = 2) were identified as COMES and EQ-MEs. These are qualitative instruments developed by researchers to evaluate menus regarding the country’s national recommendations. Most of the studies have a qualitative approach (*n* = 13, 81.25%), and only 31.25% (*n* = 5) of the studies present quantitative data (Table 1).

The studies by Da Silva Bastos Soares et al. [13], Vidal et al. [16], and Longo-Silva [14] presented the Qualitative Evaluation of Menu Preparation (QEMP) as a method of qualitative evaluation of menus using variables related to food and culinary method.

The study by Moreira Sampaio [15] brought a quantitative approach. It sought to assess the portions’ nutritional composition, also evaluating the differences between the offered menu and the planned menu. As a result of this study, two school menu proposals were developed based on the nutritional recommendations of the National School Feeding Program (PNAE) [27]. A list of substitutions for handlers to meet possible menu changes was also developed.

Martins Rodrigues [2] proposed and validated a qualitative checklist instrument to evaluate school menus, emphasizing nutritional, sanitary, and sustainability aspects. Based on Brazilian legislation, an instrument with 136 questions was developed, which includes 62 items divided into eight blocks for sanitary hygienic aspects, physical structure, types of equipment, and utensils; 36 items divided into four blocks for nutritional aspects, school menus, and nutritional recommendations for schoolchildren; and 38 items with approaches to sustainability and good environmental practices. There are three possible answers for each item: yes (adequate), no (inadequate), and does not apply. At the end of the checklist’s application, the menu and the school food service are evaluated as excellent, good, regular, bad, and terrible. The percentages of adequacy were: excellent—81 to 100; good—61 to 80; regular—41 to 60; bad—21 to 40; and terrible—0 to 20.

Studies in Spain focused on developing researchers’ methods for qualitative evaluation of school menus’ nutritional balance, meeting national recommendations [17,18,19,21].

Among the instruments developed in Spain, the COMES questionnaire [17] and the EQ-MEs [19] qualitatively evaluate school menus. COMES includes a table with 15 items. The first eight assess the frequency of food groups. The other seven assess the menus’ general characteristics such as fried food, various preparations/ dishes, food preferences, menu rotation, and type of fat used.

The EQ-MEs complements the COMES, with a list of 17 items, specifying food groups and culinary methods, based on recommendations from the Spanish government and a panel of experts. The EQ-MEs assess the quality of school menus and their suitability regarding nutritional aspects in the child population when compared to national recommendations [17,19].

Recently, Soares et al. [20] developed a study in Spain to evaluate the implementation of the Consensus Document on Food in Educational Centers (DCSECE), which includes a minimum set of indicators for the evaluation and follow-up of nutritional recommendations that serve to guide menu planning and how school canteens must respond.

Evans and Cade [24] developed a Quality Index (ranging from 0–21) as a dietary assessment method with nutritional calculation. The portions were weighed, evaluated, and scored using a list of thirteen nutrients and eight foods (five healthy and three restricted/unhealthy). The studies by Myers [7], Dubuisson [23], and Therre et al. [22] emphasize the variety of studies with a qualitative evaluation of school menus, and the difficulty of extending this evaluation due to lack of data, little involvement of the nutrition professionals in planning menus, and different realities in school environments and public policies.

As for the evaluation criteria (nutritional, sensory, cultural, and sustainability), the nutritional criterion was identified in all studies (100%), followed by the sensorial criterion (*n* = 6, 37.5%) and the sustainability criterion (12.5%). No study evaluated the cultural criterion. None of the studies presented three different forms of evaluation criteria.

### 3.3. Bias Assessment

The studies are heterogeneous, but most, 93.75% (*n* = 15), presented a low risk of bias, and one presented a moderate risk of bias (Table S3—Supplementary File). All studies evaluated specific methods of evaluating school menus and answered the main question (Table S3—Supplementary File).

## 4. Discussion

This systematic review is the first to analyze how school menus are evaluated in some countries. The results showed that Brazil and Spain were the countries that presented the largest number of studies evaluating menus. In Brazil, adequate and healthy food is a guaranteed right for students enrolled in public, philanthropic, and community organizations, in partnership with the government and guaranteed by the National School Feeding Program. This is characterized as a long-term public policy in Brazil for food and nutritional security and is considered one of the largest, most comprehensive, and long-term programs in the area of school meals globally [28].

In Brazil, the increase in the number of overweight and obese children [29] and the changes in the population’s lifestyle and eating habits resulting from growing urbanization reinforce the school’s important role in healthy eating habits and health promotion. The school is an environment for health promotion and actions on food and nutritional education [14]. These changes and the consolidation of public policies for schoolchildren may justify the number of studies aimed at evaluating the school menu. However, none of the studies presented instruments or methods for evaluating the four menu planning components.

Spain has one of the highest prevalence of overweight and obesity in Europe. Epidemiological studies show that 26.2% of children aged 6–9 are overweight, and 18.3% obese [21]. In 2005, a consensus on school feeding was published in Spain. This NAOS strategy (Nutrition, Physical Activity, and Prevention of Obesity) focused on healthy eating habits in the school environment and physical activity to fight obesity. Thus, a consensus document on food in educational centers (DCSECE) was defined. This contains a minimum set of indicators for the evaluation and follow-up of nutritional recommendations that guide menu planning and how school canteens should serve these menus [18,20,21,30].

In the NAOS strategy context, Spain consolidates itself with legal guidelines for developing healthier school menus. Several organizations and entities in Spain have developed systems for the supervision and qualitative assessment of school menus based on food groups, type of fat, and fiber and sugar [17]. There is a need to improve evaluation with a quantitative approach.

The other studies, unique among the countries of Europe and Oceania, present various methods of evaluating school menus proposed by researchers to meet national recommendations in the context of school meals. These include a frequency table with comparison to national recommendations, a table of food groups compared to national recommendations, a food group table correlated with a traffic light system, and a quality index of school meals compared to recommendations. The approaches are qualitative and assess the nutritional aspect exclusively, comparing these to nutritional recommendations [7,22,23,24,26].

Most studies, 81.25% (*n* = 13), presented methods for assessing qualitative data on school menus, i.e., the evaluation of frequency, variety, and quality of food, compared to the nutritional recommendations of the country of the study. These studies show the difficulty of extending evaluation due to lack of data about composition and preparation technique or from the Technical Preparation File (TPF). TPF is an instrument for promoting health, based on specifying culinary preparations with registration of the components and their quantities [31]. It also contains registration of the culinary techniques, the direct and indirect cost, nutrients, and other information for the food and nutrition service. From the TPF, it is possible to estimate the nutrients in the recipe and the portion, to guarantee standardization, and to modify possible misfits. Thus, in the absence of TPF, it is not feasible to accurately assess which nutrients make up a recipe [31]. Added to these difficulties are the scarce involvement of dietitians in menu planning and the various different realities in school environments when dealing with public policies [7,18,22,23,30].

The Qualitative Assessment of Menu Preparations (Avaliação Qualitativa das Preparações do Cardápio—AQPC) [32] is an instrument of menu management to propose balanced meals. It allows the assessment of the nutritional balance and sensory aspects of the menu, considering colors, preparation techniques, repetitions, combinations, the offer of certain foods such as fruits, vegetables, or types of meat, in addition to the sulfur content of the food [32]. The focus is qualitative. The instrument fails to reach all the Brazilian school feeding program recommendations, which presents quantitative, cultural, and sustainability parameters that must be followed in all municipalities in the country [13,14,16].

The checklist instrument proposed by Martins Rodrigues [2] states that school menus need to attend to nutritional, hygienic-sanitary and sustainability aspects. The instrument brings a broad approach to all the inherent aspects of Food and Nutrition security. It is not exclusive to the evaluation of school menus and also addresses aspects of the physical structure of the food production area. The checklist allows for the registration of schools, for the automatic calculation of the percentage of the evaluated blocks’ adequacy, and for reassessments and long-term monitoring. With this assessment and its approaches illustrating limitations, the school meal service becomes an appropriate environment focusing on health, encompassing nutritional aspects and sanitary and environmental aspects.

Quantitative data studies [15,24,25] present nutritional calculation for some nutrients such as macronutrients, iron, and vitamin D but do not assess consumption. The served portion is evaluated and not the consumed portion. These studies quantitatively assess the nutritional aspects of school menus and their adequacy given nutritional recommendations.

According to Moreira Sampaio [15], quantitative and qualitative evaluation is essential for students’ growth and development and eating habits that will influence adult patterns. Integrating all these aspects is needed to respect the right to healthy food established by the ONU. From this perspective, school feeding seeks to contribute so that food and nutritional security are guaranteed for all.

There is a lack of studies from North America, Central America, Africa, and Asia regarding menu evaluation instruments or methods. Despite this, the USA has two school feeding programs: the NLSP (National Lunch School Program) and the SBP (School Breakfast Program) to ensure that children can access healthy balanced meals within schools. As in other countries, in the USA, 31.8% of children and adolescents aged 2–19 years are overweight or obese [33]. According to Belik [34], America has almost 20 counties with School Meal programs; however, many programs only give small grants to needy schools. The USA presents programs, but no instrument or method to evaluate the menus reported in the literature [34].

In the world scenario during the COVID−19 pandemic, this is even a more evident need. Even before the Pandemic, a FAO [35] publication reveals that approximately 690 million people worldwide, the equivalent of 8.9%, were malnourished. The consequences experienced by the most vulnerable populations in the pandemic, such as the interruption of food supply and family income reduction, make access to adequate food even more distant.

This new scenario reinforces the need to monitor and evaluate school feeding. In this way, it will be possible to stimulate initiatives that respect the recommendations to promote adequate and healthy food. The intended result is to decrease malnutrition and reverse the negative trends of increasing obesity and other chronic diseases related to food. It is crucial to seek this in order to meet what is advocated as a healthy diet, that derives from an adequate nutritional supply and a socially and environmentally sustainable food system [35].

According to Maynard et al. [36], sustainability integrates actions based on three pillars: environmental, social, and economic. Its focus is on the pursuit of quality of life and environmental balance [36]. Besides, Ginani et al. [4] reinforce the importance of using regional foods as a sustainable way of promoting health. Therefore, a greater significance of food is confirmed, which goes beyond the biological and establishes it as a social and cultural act. Food is then an identity marker [4]. However, none of the analyzed methods and instruments covered the cultural aspect, including regionality, and only 12.5% (*n* = 2) observed the menu’s sustainability. This fact shows a gap in the field that must be quickly filled, especially facing world events that affect all sustainability dimensions.

In addition to hunger, which causes a great social impact in itself, the environment has suffered from long-term natural and human-made events [37]. The impact of these aspects on the economy is noted in several sectors. Lost natural resources and increased public health costs directly affect the development of areas that are no longer priorities. All these aspects justify the need to value sustainable food systems and include them in planning menus aimed at the community. In the case of children, it is possible to learn about different topics in a transversal way. This also adds to what is recommended as healthy eating.

The studies show the problems that make it impossible for schools to serve their planned menus, failure to deliver goods, the degree of ripeness of fruits and vegetables, and the planned menus’ low acceptability. In this context, the need for the dietitian’s presence in the school environment is reinforced, playing an essential role in nutritional education and developing TPFs to guarantee adequate nutritional recommendations and minimize the school’s negative impacts on the menus.

There are numerous dietary guidelines for proper planning of school menus, but a nutritional assessment of these menus is still a challenge [14,38]. Progress has been made to harmonize the menus provided in schools with standards based on scientific research and nutritional recommendations to achieve adequate nutrition. However, an effort is still needed to plan and evaluate school meals to meet all nutritional requirements, composition, variety, conservation methods, diverse culinary techniques, origins and ethnicities, allergies and intolerances, and hygienic-sanitary standards [39].

The menu stands out as an essential working tool to achieve nutritional recommendations. Planning the menu also contributes to achieving sensory, cultural, and sustainability aspects. Thus, it is relevant to understand the planning of school meals, from food acquisition to final distribution, considering the variables between who buys and distributes. The nutrition professional must follow and participate in the whole process. They should understand the seasonality of products, respect the mapping of farmers’ production to ensure the supply of fruits and vegetables systematically, and monitor on-site the stages of meals’ preparation and proper distribution [13,18,28].

Few studies present tools for evaluating menus, which, in turn, are mostly based on nutritional criteria. A global assessment of the menu is necessary to offer healthy meals. Thus, the school menu assessment instrument should include qualitative and quantitative aspects, crossing over hygienic and sanitary, cultural and sustainability issues. When planning the menu, using all these tools will allow a careful assessment to meet the proposed objectives.

This review has some limitations since the studies from different countries were collected on the mentioned platforms and, despite the abstract being in English, some studies were written in another languages. Despite the usage of a translation platform, some data could have been missed due to language barriers. There could also be school feeding programs that present instruments or methods to evaluate their menus, but were not published before the moment of this review search on databases.

## 5. Conclusions

There is a significant opportunity to serve healthy and tasty meals in school food services, to possibly compensate for the user population’s daily nutritional deficiencies. Considering that students spend most of their day at school, eating up to three meals, it is imperative to control and evaluate school menus. This is the only way to guarantee healthy eating habits, various food groups, safe food from the hygienic-sanitary point of view, and respect for food and cultural habits. However, the present study did not identify an instrument for evaluating school menus that contemplates a comprehensive approach to aspects that involve adequate nutrition.

National school feeding programs should provide the evaluation stage as a decisive part of planning menus. Countries such as Brazil and Spain, where a more significant number of instruments have been identified, should serve as models to achieve school feeding goals for all students.

Menu planning and evaluation must cover nutritional aspects and cultural, sensory, sanitary, and sustainable principles. School feeding programs should encourage the development of instruments and/or methods for evaluating menus to help dietitians better plan menus for students.

Other search methodologies should be applied to identify school feeding programs and search for instruments not published in the literature. Specific school feeding programs probably present other instruments or methods to evaluate their menus that are not available in the literature. A search of nation’s websites regarding school feeding programs could reveal how menus are developed and evaluated, gathering more information on other methods not scientifically described in the literature. Dietitians around the world could be developing or using different methods that could enrich this field of study. Thus, it is planned to gather other experiences in other countries not covered in this research. The current results show a lack of menu evaluation and this could contribute to a population’s nutritional inadequacy.

## Figures and Tables

**Table 1:** Characteristics of the studies included in the systematic review regarding menu planning evaluation.

Year	Authors	Country	Study Design	Subject	Type of Protocol		Specific Name		Presence of Evaluation Criteria				Type of Data	
					Instrument	Method	Yes	No	Nutritional	Sensorial	Cultural	Sustainability	Qualitative	Quantitative
2015	De Mateo Silleras et al. [17,18,19,20,21,22]	Spain	Prospective longitudinal study	School menu	X		COMES		YES	NO	NO	NO	X	
2009	Dubuisson et al. [23]	France	Descriptive, cross-sectional study	Elementary school and high school		X		X	YES	NO	NO	NO	X	
2017	Evans and Cade [24]	England	Descriptive, cross-sectional study	Scholars from 08 to 09 years		X		X	YES	NO	NO	NO		X
2018	Llorens-Ivorra et al. [19]	Spain	Observational, cross-sectional study	Primary school (6 to 14 years)	X		EQMES		YES	YES	NO	NO	X	
2013	Patterson et al. [25]	Sweden	Experimental study	Primary school	X			X	YES	NO	NO	YES		X
2020	Martins Rodrigues et al. [2]	Brazil	Descriptive and transversal study	Pre-school	X			X	YES	NO	NO	YES	X	
2013	Longo-Silva et al. [14]	Brazil	Experimental study	Public and private schools		X		X	YES	YES	NO	NO	X	X
2015	Gregoric et al. [26]	Slovenia	Descriptive, cross-sectional study	Primary school (6 to 14 years)		X		X	YES	NO	NO	NO	X	X
2018	González et al. [18]	Spain	Descriptive, cross-sectional study.	Primary school (6 to 14 years)		X		X	YES	YES	NO	NO	X	
2019	Myers et al. [7]	Australia	Descriptive, cross-sectional study.	Public and private schools		X		X	YES	NO	NO	NO	X	
2015	Da Silva Bastos Soares et al. [13]	Brazil	Descriptive, cross-sectional study.	School menus		X		X	YES	YES	NO	NO	X	
2017	Moreira Sampaio et al. [15]	Brazil	Cross-sectional study	School menus		X		X	YES	NO	NO	NO		X
2015	Vidal et al. [16]	Brazil	Cross-sectional study	School menus		X		X	YES	YES	NO	NO	X	
2015	Uriarte et al. [21]	Spain	Descriptive cross-sectional study	School menus		X		X	YES	NO	NO	NO	X	
2019	Soares et al. [20]	Spain	Transversal study	School menus		X		X	YES	YES	NO	NO	X	
2012	Therre et al. [22]	Germany	Transversal study	School menus		X		X	YES	NO	NO	NO	X	

## Data Availability

Not applicable.

## Supplementary Materials

The following are available online at https://www.mdpi.com/2304-8158/10/2/374/s1. Table S1: Full-text excluded articles and reasons, Table S2: Indexers used to select publications that jointly or separately address instruments and methodologies for evaluating school menus, Table S3: The summarized risk of bias assessment of the studies included in this systematic review.
