# Supplementary material for: How Are School Menus Evaluated in Different Countries? A Systematic Review
Source: Foods. 2021 Feb 9;10(2):374. doi: 10.3390/foods10020374 (PMC7915021; doi:10.3390/foods10020374)
Supplement: Supplementary file 1 [file foods-10-00374-s001.pdf]

foods

MDPI

Supplementary Materials

Table S1. Full-text excluded articles and reasons.

Author (year)	Reference	Exclusion Motif
Camargo et al. (2017)	[1]	1
Aranha et al. (2017)	[2]	1
Laval et al. (2020)	[3]	2
Armel et al. (2019)	[4]	2
Serrem et al. (2020)	[5]	3
Byker et al. (2013)	[6]	4
Teo (2017)	[7]	4
Laar & Laar (2017)	[8]	1
Galante et al. (2017)	[9]	1
Chichizola et al. (2017)	[10]	1
Owusu et al. (2016)	[11]	2
Castro et al. (2016)	[12]	2
Retondario et al. (2016)	[13]	2
Issa et al. (2014)	[14]	4
Longo-Silva et al. (2013)	[15]	2
Menegazzo et al. (2011)	[16]	2
Campos et al. (2011)	[17]	1
Da Cunha et al. (2014)	[18]	2
De Castro et al. (2014)	[19]	2
Flavio et al. (2008)	[20]	3
Flavio et al. (2004)	[21]	3
Bertin et al. (2012)	[22]	2
Burghardt (1995)	[23]	2
Campos-Diaz et al. (2008)	[24]	2
Clark e Fox (2009)	[25]	2
Crepinsek et al. (2009)	[26]	3
Da Silva (1995)	[27]	2
França et al. (2018)	[28]	3
Gearan & Fox (2020)	[29]	4
Gougeon et al. (2011)	[30]	2
Martinez et al. (2010)	[31]	3
Richter et al. (2012)	[32]	2
Seiquer et al. (2015)	[33]	3
Zulueta et al. (2011)	[34]	2

Legend – Exclusion criteria: 1 -) comments, letters, conference, review, abstracts, papers and books (n=6); 2 - studies that assessed consumption and not in the planning stage (n=17); 3 - studies that evaluated the portion and nutritional composition (n=7); 4 studies that address the guidelines but do not evaluate the menu (n=4).

Table S2. - Indexers used to select publications that jointly or separately address instruments and methodologies for evaluating school menus.

**PUBMED 294**	**(((("school meal" OR "school meals" OR "school menu" OR "school menus" OR "school food service" OR "school food services")) OR ("school feeding program" OR "school feeding programs"))) AND ("evaluation" OR "assessment")**
**LILACS 14**	("school meal" OR "school meals" OR "school menu" OR "school menus" OR "school menu planning" OR "school food service" OR "school food services" OR “menu planning”) AND ("school feeding program" OR "school feeding programs")
**WEB OF SCIENCE**	TS=(“school feeding programs” OR “school feeding”) AND TS=("school meals" OR "school menu evaluation" OR “evaluation” OR "assessment")

**145**	
**SCOPUS 251**	("school meal" OR "school meals" OR "school menu" OR "school menus" OR "school menu planning" OR "school food service" OR "school food services") AND ("school feeding program" OR "school feeding programs") AND ("evaluation" OR "assessment")
**SCIENCE DIRECT 457**	("school meal" OR "school menu" OR "school menu planning" OR "school food service" OR ("school feeding program") AND (evaluation OR assessment)
**PROQUEST 1828**	(School feeding programs) AND (School Meals) AND (school menu evaluation) From 2015 to 2020
**GOOGLE SCHOLAR 200 more relevant 640 results**	*allintitle (School feeding program OR school feeding OR school meal OR school menus) and (school menu planning OR menu evaluation)*

References

1. Camargo et al. What is the quality of school meals in brazil? **2017**, 264–271.
2. Aranha et al. Evaluation of the menus offered to preschool children in the city of botucatu. SP. in; 2017.
3. Lavall, M. J. nutritional assessment of the school menus offered in Spain's mediterranean area. **2020**.
4. Armel, Evaluation of the nutritional quality of the menus served in the communal canteens of a school and a high school in the Valencian Community. **2019**.
5. Serrem, K.; Dunay, A.; Serrem, C.; Atubukha, B.; Oláh, J.; Illés, C. B. Paucity of nutrition guidelines and nutrient quality of meals served to kenyan boarding high school students. *Sustain.* **2020**, *12*, 1–12, doi:10.3390/SU12083463.
6. Byker, C. J. New NSLP Guidelines: Challenges and Opportunities for Nutrition Education Practitioners and Researchers. **2013**.
7. Teo, C. R. P. A. The partnership between the Brazilian School Feeding Program and family farming: a way for reducing ultra-processed foods in school meals. **2017**.
8. Laar and Laar. School feeding program implementation and child nutrition in ghana: improved methods and tools. 2017.
9. Galante et al Initial situation assessment on school food and nutrition in africa. In; 2017.
10. Chichizola, et al. Evaluation of a school feeding program in a real setting. In; 2017.
11. Owusu, J. Comparison of Two School Feeding Programmes in Ghana, West Africa. **2016**.
12. Castro, M.; Ríos-Reina, R.; Ubeda, C.; Callejón, R. M. Evaluación de menús ofertados em comedores escolares: comparación entre colegios públicos, privados y concertados / Evaluation of school menus®: Comparing public, private and charter schools. *Rev. Nutr.* **2016**, *29*, 97–108, doi:10.1590/1678-98652016000100010.
13. Retondario, A.; Silva, D. L. F.; Salgado, S. M.; De Oliveira Alves, M. A.; Ferreira, S. M. R. Nutritional composition of school meals serving children from 7 to 36 months of age in municipal day-care centres in the metropolitan area of Curitiba, Paraná, Brazil. *Br. J. Nutr.* **2016**, *115*, 2203–2211, doi:10.1017/S0007114516001434.
14. Issa, R. C.; Moraes, L. F.; Francisco, R. R. J.; Santos, L. C. dos; Anjos, A. F. V. dos; Pereira, S. C. L. *Revista panamericana de salud publica.*; Organizacion Panamericana de la Salud, 2014; Vol. 35;.
15. Longo-Silva, G.; Toloni, M.; Rodrigues, S.; Rocha, A.; Taddei, J. A. de A. C. Qualitative evaluation of the menu and plate waste in public day care centers in são paulo city, brazil. *Rev. Nutr.* **2013**, *26*, 135–144, doi:10.1590/S1415-52732013000200002.
16. Menegazzo, M. Qualitative evaluation of the foods served in children education centers Menegazzo et al 2016_supplementary data. **2011**.
17. Campos et al. Quality evaluation of school feeding in public pre-school in Brazil. In; 2011.
18. da Cunha, D. T.; Gonçalves, H. V. B.; de Lima, A. F. A.; Martins, P. A.; de Rosso, V. V.; Stedefeldt, E. Regional food dishes in the Brazilian national school food program: Acceptability and nutritional composition. *Rev. Nutr.* **2014**, *27*, 423–434, doi:10.1590/1415-52732014000400004.
19. De Castro, T. G. Management, operations, and menus in Kaingáng indigenous schools participating in the National School Nutrition Program in Rio Grande do Sul State, Brazil. **2014**.
20. Flávio, E. F.; Barcelos, M. de F. P.; Lima, A. L. de Avaliação química e aceitação da merenda escolar de uma escola estadual de Lavras-MG. *Ciência e Agrotecnologia* **2004**, *28*, 840–847, doi:10.1590/s1413-70542004000400016.
21. Flávio, E. F.; Barcelos, M. de F. P.; Lima, A. L. de EvaluationofschoolmealsofferedtoelementarystudentsofmunicipalschoolsofLavras,MG. **2008**.
22. Bertin, M. School meals in French secondary state schools: Do national recommendations lead to healthier nutrition on offer? Compatibilidade com o leitor de tela ativada. **2012**.
23. Burghardt, J. A. School Nutrition Dietary Assessment Study: Overview of the study design. *Am. J. Clin. Nutr.* **1995**, *61*, 182–186, doi:10.1093/ajcn/61.1.182s.
24. Campos Díaz, J. Valoración nutricional de los menús escolares de los colegios públicos de la isla de Tenerife. **2008**.

25. Clark e Fox Nutritional Quality of the Diets of US Public School Children and the Role of the School Meal Programs. **2009**.
26. Crepinsek, M. K.; Gordon, A. R.; McKinney, P. M.; Condon, E. M.; Wilson, A. Meals Offered and Served in US Public Schools: Do They Meet Nutrient Standards? *J. Am. Diet. Assoc.* **2009**, *109*, S31–S43, doi:10.1016/j.jada.2008.10.061.
27. Silva, M. V. da Avaliação da Adequação Nutricional dos Alimentos Consumidos em um Centro Integrado de Educação Pública (CIEP). **1995**.
28. França, F. C. O. School meals' centesimal and mineral composition and their nutritional T value for Brazilian children. **2018**.
29. Gearan, E. C.; Fox, M. K. Updated Nutrition Standards Have Significantly Improved the Nutritional Quality of School Lunches and Breakfasts. *J. Acad. Nutr. Diet.* **2020**, *120*, 363–370, doi:10.1016/j.jand.2019.10.022.
30. Gougeon, L. A. R.; Henry, C. J.; Ramdath, D.; Whiting, S. J. Dietary analysis of randomly selected meals from the Child Hunger and Education Program School Nutrition Program in Saskatchewan, Canada, suggests that nutrient target levels are being provided. *Nutr. Res.* **2011**, *31*, 215–222, doi:10.1016/j.nutres.2011.03.002.
31. Martínez, A. B.; Caballero-Plasencia, A.; Mariscal-Arcas, M.; Velasco, J.; Rivas, A.; Olea-Serrano, F. Estudio de los menús escolares servidos en colegios de Granada. *Nutr. Hosp.* **2010**, *25*, 394–399, doi:10.3305/nh.2010.25.3.4294.
32. Richter, S. L.; Vandervet, L. M.; Macaskill, L. A.; Salvadori, M. I.; Seabrook, J. A.; Dworatzek, P. D. N. Accuracy and Reliability of Direct Observations of Home-Packed Lunches in Elementary Schools by Trained Nutrition Students. *J. Acad. Nutr. Diet.* **2012**, *112*, 1603–1607, doi:10.1016/j.jand.2012.07.010.
33. Seiquer, I.; Haro, A.; Cabrera-Vique, C.; Muñoz-Hoyos, A.; Galdó, G. Evaluación nutricional de los menús servidos en las escuelas infantiles municipales de Granada. *An. Pediatr.* **2016**, *85*, 197–203, doi:10.1016/j.anpedi.2015.07.020.
34. Zulueta et al Nutritional profile of foods offered and dietary intake in school canteens in Biscay. In; 2011.
